# Coadministration of *Pinellia ternata* Can Significantly Reduce *Aconitum carmichaelii* to Inhibit CYP3A Activity in Rats

**DOI:** 10.1155/2014/734867

**Published:** 2014-10-14

**Authors:** Jinjun Wu, Zaixing Cheng, Lijun Zhu, Linlin Lu, Guiyu Zhang, Ying Wang, Ying Xu, Na Lin, Zhongqiu Liu

**Affiliations:** ^1^Institute of Chinese Meteria Medica, China Academy of Chinese Medical Sciences, Beijing 100700, China; ^2^International Institute for Translational Chinese Medicine, Guangzhou University of Chinese Medicine, Guangzhou 510006, China; ^3^College of Pharmacy, Fujian University of Traditional Chinese Medicine, Fuzhou 350108, China

## Abstract

Chuanwu (CW), the mother root of *Aconitum carmichaelii* Debx., is a traditional Chinese medicine (TCM) for treating traumatic injuries, rheumatoid arthritis, and tumors. CW coadministered with banxia (BX), the root of *Pinellia ternata*, is also widely prescribed in clinical practice. However, the mechanism of this combination is yet deciphered. Current study aimed to investigate the effects of CW, including raw chuanwu (RCW) and processed chuanwu (PCW) alone, as well as CW coadministered with BX on CYP3A activity. Buspirone (BP) and testosterone (Tes) were used as specific probe substrates *in vivo* and *ex vivo*, respectively. CYP3A activity was determined by the metabolites formation ratios from the substrates. Compared with those in the control group, the metabolites formation ratios significantly decreased in the RCW and PCW alone groups, accompanied by a marked decrease in CYP3A protein and mRNA levels. However, there was a significant increase in those ratios in the RCW-BX and PCW-BX groups compared to the RCW and PCW alone groups. The results indicated that both RCW and PCW can inhibit CYP3A activity in rats because of downregulation of CYP3A protein and mRNA levels. Decreases in CYP3A activity can be reversed by coadministration with BX.

## 1. Introduction

Chuanwu (CW), the mother root of* Aconitum carmichaelii *Debx., includes raw chuanwu (RCW) and processed chuanwu (PCW). CW is used only after processing because of its high toxicity, which results in side effects and adverse clinical reactions, such as severe arrhythmia and neurotoxicity [1–3]. Nowadays, CW is used widely in China and other Asian countries for rheumatoid arthritis, cardiovascular diseases, tumors, and diarrhea [4–7].

Banxia (BX), the tuber of* Pinellia ternata* (Thunb.) Breit., is often coadministered with CW in many clinical formulae for toxicity reduction and pharmacological effect improvement. According to statistics, over 400 prescriptions that contain both CW and BX, such as* Chiwan Fang*,* Fuzi Nuomi Tang*, and* Wutou Banxia San*, are recorded in* Zhongyi Fangji Da Cidian *(which describes over 100,000 classic prescriptions recorded through the centuries) [8]. Despite the wide coadministration of the two herbs, the exact mechanism of their combined effects has yet to be explored thoroughly.

Many studies have investigated the combined effects from the perspective of compound interactions between the two herbs and agree that BX alters the content of diester diterpene alkaloids, which are the pharmacologically active and toxic ingredients of CW that result in its efficacy and toxicity. For example, a previous study reported significant differences in chemical ingredients in BX-CW water extracts with different combination ratios. Along with the increasing proportion of BX, the content of diester diterpene alkaloids decreased gradually [9, 10]. Water extracts containing raw BX inhibit the conversion of diester alkaloids and increase their content in extracts [11–13]. Unfortunately, no studies are yet available on the underlying compatibility mechanism of the two herbs.

CYP3A, the most abundant P450 enzyme in the liver, is involved in the metabolism of 40% to 50% of all currently used drugs [14]. However, CYP3A activity is frequently affected by its own substrates and is recognized by authorities as one of the most important causes of drug-drug interaction (DDI) between herbal medicines and prescription drugs. Clinical trials have confirmed that common herbal medicines, including St. John's wort (*Hypericum perforatum*), ginkgo (*Ginkgo biloba*), ginger (*Zingiber officinale*), ginseng (*Panax ginseng*), and garlic (*Allium sativum*), interact with drugs frequently. For example, St. John's wort (*Hypericum perforatum*) significantly reduces the area under the plasma concentration-time curve (AUC) and blood concentrations of cyclosporine, midazolam, tacrolimus, amitriptyline, digoxin, indinavir, warfarin, phenprocoumon, and theophylline via CYP3A and/or P-glycoprotein induction [15]. In clinics, CW is frequently used concomitantly with other herbal medicines or prescription drugs for treating complex diseases. Thus, the safety assessment of CW-related multiple drug therapy is important. Nevertheless, few studies have reported the influence of CW on CYP3A activity. The variations in CYP3A activity after the coadministration of BX also remain unclear. An interaction study about the cotherapy of the two herbs from the perspective of CYP3A activity is thus urgent.

In the current study, CYP3A activity* in vivo *and* ex vivo* was examined after rats were pretreated with CW alone or jointly with BX for 7 d. Probe substrates are commonly used to evaluate the effects of target drugs on CYPs. We choose buspirone (BP) as the specific probe substrate for CYP3A activity* in vivo* according to the US Food and Drug Administration (USFDA, 2006). BP is primarily metabolized by CYP3A into 1-(2-pyrimidinyl) piperazine (1-PP) and 6′-hydroxybuspirone (6′-OH-BP) [16]. The pharmacokinetic behavior of BP after pretreatment with CW or CW-BX water extracts was investigated. The plasma concentrations of BP, 1-PP, and 6′-OH-BP were determined using a validated ultra-performance liquid chromatography-tandem mass spectrometry (UPLC-MS/MS) method. Testosterone (Tes) was chosen as the specific probe substrate to examine the impact of CW or CW-BX on CYP3A activity in rat liver microsomes (RLMs) by measuring the formation rate of its metabolite 6*β*-hydroxytestosterone (6*β*-OH-Tes) by a UPLC method [17, 18]. To further explore the molecular mechanisms of the changes in CYP3A activity, CYP3A protein and mRNA expression levels were analyzed via western blot and real-time PCR.

## 2. Materials and Methods

### 2.1. Chemicals and Reagents

Buspirone hydrochloride (BP·HCl), Tes (purity > 98%), 6*β*-hydroxytestosterone (purity > 98%), and gemfibrozil (used as an internal standard, purity > 98%) were purchased from Sigma-Aldrich (St. Louis, MO, USA). 1-PP, 6′-OH-BP, and SKF-525A hydrochloride (used as a positive control drug) were obtained from Toronto Research Chemicals, Inc. (Toronto, CA). An NADPH regenerating system was purchased from BD Gentest Corp. (Woburn, MA, USA). HPLC-grade acetonitrile for liquid chromatography was purchased from Merck (Darmstadt, Germany). Deionized water was prepared by a Millipore Milli-Q Plus system (Millipore Bedford, MA, USA). All other chemicals used were of analytical reagent grade or better.

### 2.2. Plant Material

*A. carmichaelii* Debx. (RCW and PCW) and* P. ternate* (Thunb.) Breit. (BX) were purchased from Huamiao Traditional Chinese Medicine Engineering Technology Development Center (Beijing, China) and confirmed by Institute of Chinese Materia Medica, China Academy of Chinese Medical Sciences.

### 2.3. Animals

Male Sprague-Dawley rats weighing 180–220 g were supplied by the Laboratory Animal Center of Guangzhou University of Chinese Medicine (Guangzhou, China; License: SCXK (yue) 2008-0020). The rats were housed in a unidirectional airflow room under controlled temperature (23°C), relative humidity (40%–70%), and a 12 h light/dark cycle with free access to standard rat food and water. All animal treatments followed guidelines for the care and use of laboratory animals and were approved by the Ethics Committee of Southern Medicine University (Guangzhou, China).

### 2.4. Preparation of Herbs

#### 2.4.1. Water Extracts of CW

RCW or PCW (36 g) was immersed in 720 mL of water for 30 min and then extracted with boiling water for 30 min. The residue was again extracted with 360 mL of boiling water for 60 min. The supernatant was combined and condensed to 600 mL. The ingredients in water extracts were determined via UPLC/MS/MS, showing that these extracts mainly contained monoester diterpenoid alkaloids, including benzoylaconine, benzoylmesaconine, and benzoylhypaconine. A small amount of diester diterpenoid alkaloids, such as aconine, mesaconine, and hypaconine, was also detected (data not shown).

#### 2.4.2. Water Extracts of CW-BX (1 : 1)

RCW or PCW (36 g) was immersed in 720 mL of water for 30 min and then extracted with boiling water for 30 min. BX (36 g) was immersed in another 720 mL of water for 30 min and then mixed with the RCW or PCW extracts. The mixed herbs were extracted with boiling water for 30 min. Finally, the residue was again extracted with 720 mL of boiling water for 30 min. The two batches of filtrates were combined and condensed to 600 mL. All water extract samples were refrigerated at −20°C. The samples were warmed in 37°C water baths before administration to rats by gavage. The ingredients in water extracts were determined via UPLC/MS/MS. The monoester diterpenoid alkaloids from CW were detected; and trigonelline, which was the major bioactive ingredient from BX, was also detected (data not shown).

### 2.5. Pharmacokinetics of BP in Rats with 7 d CW or CW-BX Water Extract Pretreatment

#### 2.5.1. Collection of Plasma Samples

Rats were randomly divided into five groups with five animals in each group. After pretreatment with RCW, PCW, RCW-BX, or PCW-BX water extracts (0.6 g·kg^−1^·d^−1^, p.o.) or saline for 7 d, the rats were given BP·HCl (0.5 mg·kg^−1^, single dose, i.v.). Blood samples were collected at 0, 2, 7, 15, 30, 60, 90, 120, 180, 300, 480, and 720 min after BP·HCl administration. Plasma specimens were immediately separated by centrifugation at 11,000 ×g for 8 min and stored at −20°C until analysis.

#### 2.5.2. Preparation of Plasma Samples

Rat plasma (100 *μ*L) was mixed with an aliquot (10 *μ*L) of Tes solution (6 *μ*mol·L^−1^) as an internal standard. Then, 3 × 100 *μ*L of acetonitrile was added to the mixture. The mixture was subsequently vortexed for 90 s to precipitate protein. The samples were centrifuged at 18,000 ×g for 30 min. Finally, 340 *μ*L of the supernatant was transferred to a clean tube and dried with a gentle stream of nitrogen gas. The residue was redissolved with 170 *μ*L of 95% acetonitrile aqueous solution, vortexed for 3 min, and centrifuged for 30 min at 18,000 ×g. An aliquot (10 *μ*L) of the supernatant was injected into the UPLC/MS/MS system for analysis.

### 2.6. Preparation of RLMs

Rats were randomly divided into six groups with ten animals in each group. After pretreatment with RCW, PCW, RCW-BX, or PCW-BX water extracts (0.6 g·kg^−1^·d^−1^, p.o.), saline, or SKF-525A HCl (0.1 g·kg^−1^, p.o.) for 7 d, the rats were anaesthetized with ethyl carbamate (50% w/v, 3 mL·kg^−1^, i.p.) on the eighth day, and the liver was excised. Details of the RLM preparation procedure may be found in the literature [19]. RLM samples were stored at −80°C until use. The concentration of RLM protein was determined by Bio-Rad protein assay (Bio-Rad, Hercules, CA, USA) with bovine serum albumin as the protein standard.

### 2.7. CYP3A Activity of RLMs Determined by Tes Metabolism* Ex Vivo*

The metabolite of 6*β*-OH-Tes from Tes in RLMs was evaluated by addition of various concentrations of Tes (4, 10, and 30 *μ*mol·L^−1^). All incubations were performed at 37°C in a system containing RLM protein (0.02–0.05 mg·mL^−1^) and an NADPH regenerating system at a final volume of 500 *μ*L (50 mmol·L^−1^ sodium phosphate buffer, pH 7.4). The duration of incubation was 7 min for the 4 and 10 *μ*mol·L^−1^ Tes systems and 15 min for the 30 *μ*mol·L^−1^ Tes system. Enzyme reaction was terminated by cooling on ice and addition of 4 mL of dichloromethane. The mixture was vortexed for 8 min and centrifuged at 1000 ×g for 15 min. The organic phase containing Tes and its metabolites was drawn into another tube and vacuumized. The residue was redissolved with 100 *μ*L of acetonitrile and 100 *μ*L of deionized water, and the mixture was vortexed for 3 min and centrifuged at 18,000 ×g for 30 min. The supernatant (10 *μ*L) was analyzed by UPLC. All experiments were run in triplicate.

### 2.8. CYP3A Protein Expression of RLMs by Western Blot

Microsomal protein (10 *μ*g) was loaded onto each lane and separated by SDS-PAGE (4% stacking gel, 10% separating gel). Separated proteins were transferred from the gel to the PVDF membrane. After blocking for 2 h with nonfat milk (5%, w/v) in Tris-buffered saline containing 0.1% Tween-20 (TBST), the primary antibody of rat CYP3A at 1 : 3,000 dilution (Abcam, Cambridge, UK) or *β*-actin at 1 : 1,000 dilution (Santa Cruz, USA) was added to TBST with 5% nonfat milk and incubated with the membrane at 4°C overnight. The membrane was washed and then incubated with the corresponding secondary antibody at a dilution of 1 : 4,000 in the same buffer for 1 h at room temperature. Western blot signals were obtained using ECL chemiluminescence detection agent. The relative intensity of each protein band was scanned and quantified by Quantity One (Bio-Rad, Hercules, CA).

### 2.9. CYP3A1/2 mRNA Measurement by Real-Time PCR

Rat hepatic total RNA was extracted using the TRIzol extraction method (Invitrogen, USA) according to the manufacturer's instructions. cDNA was reverse-transcribed from total RNA using a reverse transcription kit (TaKaRa, Japan). SYBR green real-time PCR amplification and detection were then performed using an ABI 7500 system (Applied Biosystems, USA). CYP3A1 primers were as follows: forward primer 5′-GGAAATTCGATGTGGAGTGC-3′ and reverse primer 5′-AGGTTTGCCTTTCTCTTGCC-3′. CYP3A2 primers were as follows: forward primer 5′-AGTAGTGACGATTCCAACATAT-3′ and reverse primer 5′-TCAGAGGTATCTGTGTTTCCT-3′ [20]. GAPDH primers were as follows: forward primer 5′-GGCCTCCAAGGAGTAAGACC-3′ and reverse primer 5′-AGGGGAGATTCAGTGTGGTG-3′. The PCR mixture had a final volume of 20 *μ*L and contained 10 *μ*L of SYBR* Premix Ex Taq* II (2x), 0.8 *μ*L each of the forward and reverse primers (10 *μ*mol·L^−1^), 2 *μ*L of cDNA, and 6 *μ*L of purified water. The thermal profile for real-time PCR was 95°C for 30 s, 95°C for 5 s, and 60°C for 34 s. A melting curve was also obtained. Target mRNA levels were normalized against GAPDH mRNA levels, and all samples were run in triplicate.

### 2.10. Analytical Conditions

#### 2.10.1. Determination of BP, 1-PP, and 6′-OH-BP* In Vivo*

The UPLC/MS/MS method used in the current study followed our previous report published in* Food and Chemical Toxicology* [16].

#### 2.10.2. Determination of Tes and 6*β*-OH-Tes* Ex Vivo*

The UPLC method used in the current study followed our previous report published in the* Chinese Journal of Experimental Traditional Medical Formulae* [21].

#### 2.10.3. Data Analysis

Pharmacokinetic parameters were determined using the standard noncompartmental method and calculated using Practical Pharmacokinetic Program Version 97 (3P97). CYP3A activity* in vivo *was determined by measuring the formation ratios of 6′-OH-BP and 1-PP from BP (6′-OH-BP/BP and 1-PP/BP ratio values) [22, 23]. CYP3A activity* ex vivo* was expressed as nanomoles of 6*β*-OH-Tes obtained per milligram of protein per minute, and results were presented as mean ± standard deviation. Significant differences were analyzed using Student's* t*-test (two groups) or one-way ANOVA followed by LSD test (for more than two groups) by SPSS 19.0. Statistical differences were considered significant at *P* < 0.05.

## 3. Results

### 3.1. Effects of RCW and RCW-BX on Pharmacokinetics* In Vivo*

**Figure 1:**
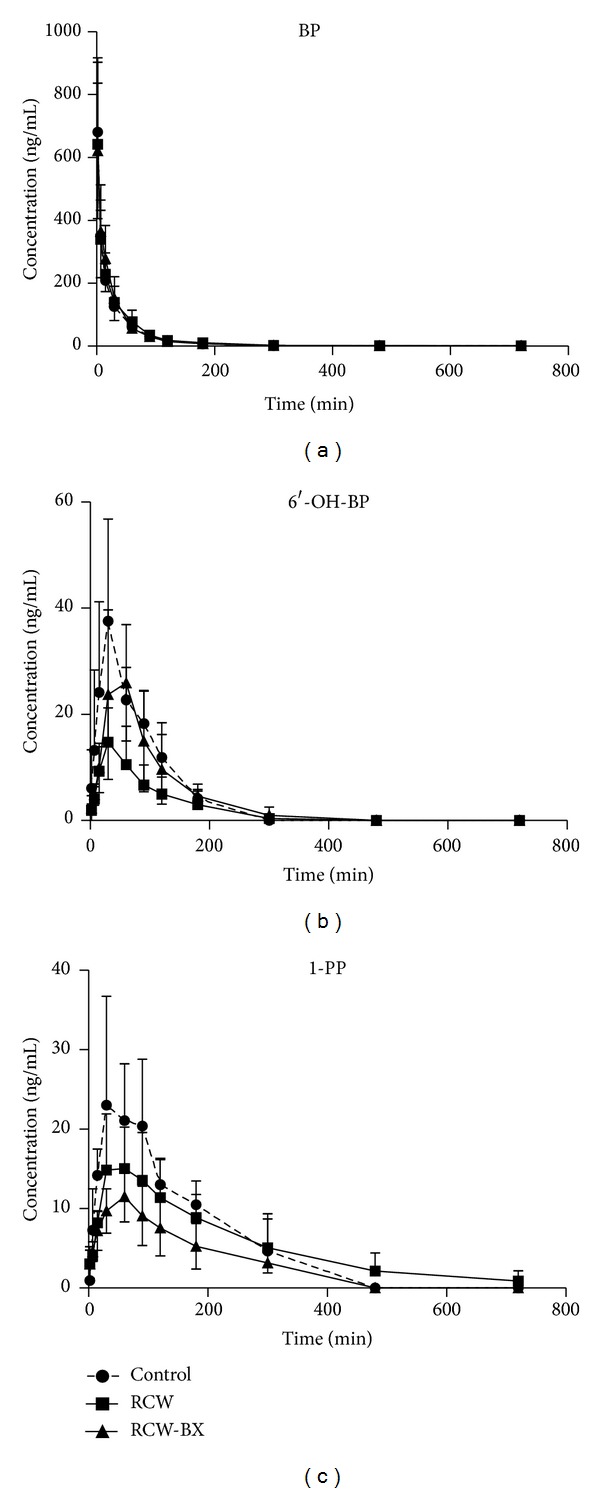
Concentration-time profiles of BP (a), 6′-OH-BP (b), and 1-PP (c) in rats after intravenous injection of 0.5 mg·kg^−1^ BP·HCl. Prior to intravenous injection of BP*·*HCl, the rats were pretreated with RCW (0.6 g·kg^−1^·d^−1^, p.o.), RCW-BX (1 : 1, 0.6 g·kg^−1^·d^−1^, p.o.), or saline once daily for 7 d. The data presented in this figure indicate mean ± SD (*n* = 5).

Prior to intravenous injection of 0.5 g·kg^−1^ BP·HCl, rats were pretreated with RCW (0.6 g·kg^−1^·d^−1^, p.o.), RCW-BX (1 : 1, 0.6 g·kg^−1^·d^−1^, p.o.), or saline once daily for 7 d. The mean plasma concentration-time profiles of BP, 1-PP, and 6′-OH-BP are shown in Figure 1, and the pharmacokinetic parameters are presented in Table 1. The formation ratio of 6′-OH-BP from BP (AUC_0-t_ of 6′-OH-BP/AUC_0-t_ of BP) in the control group was 0.24 ± 0.06, whereas those in the RCW and RCW-BX groups were 0.10 ± 0.05 and 0.18 ± 0.04, respectively. The formation ratio of 1-PP from BP (AUC_0-t_ of 1-PP/AUC_0-t_ of BP) in the control group was 0.30 ± 0.11, whereas those in the RCW and RCW-BX groups were 0.25 ± 0.08 and 0.15 ± 0.05, respectively. Compared with that in the control group, the formation ratio of 6′-OH-BP from BP decreased by 58% (*P* < 0.05) in the RCW group and increased by 80% (*P* > 0.05) after coadministration of BX. RCW also slightly decreased (*P* > 0.05) the formation of 1-PP from BP. The formation ratios of 1-PP from BP in the RCW-BX group were significantly (*P* < 0.05) lower than those in the RCW group.

### 3.2. Effects of PCW and PCW-BX on Pharmacokinetics* In Vivo*

**Figure 2:**
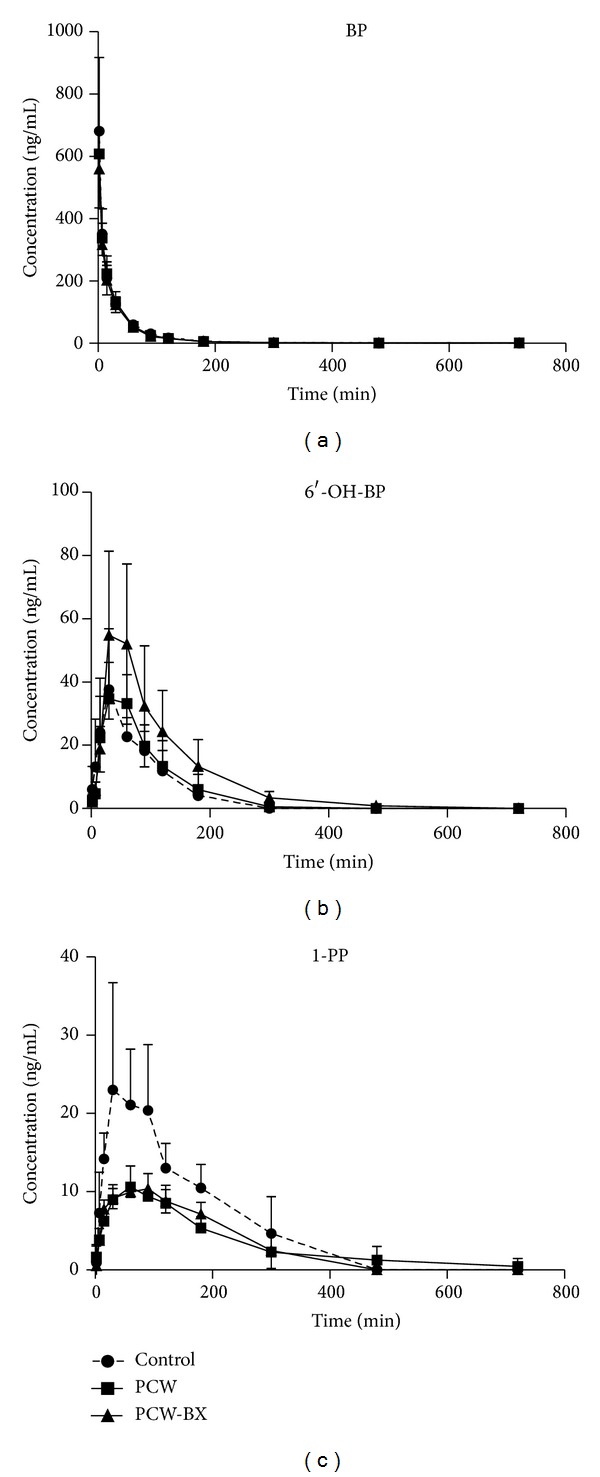
Concentration-time profiles of BP (a), 6′-OH-BP (b), and 1-PP (c) in rats after intravenous injection of 0.5 mg·kg^−1^ BP·HCl. Prior to intravenous injection of BP·HCl, the rats were pretreated with PCW (0.6 g·kg^−1^·d^−1^, p.o.), PCW-BX (1 : 1, 0.6 g·kg^−1^·d^−1^, p.o.), or saline once daily for 7 d. The data presented in this figure indicate mean ± SD (*n* = 5).

Prior to intravenous injection of 0.5 g·kg^−1^ BP·HCl, rats were pretreated with PCW (0.6 g·kg^−1^·d^−1^, p.o.), PCW-BX (1 : 1, 0.6 g·kg^−1^·d^−1^, p.o.), or saline once daily for 7 d. The mean plasma concentration-time profiles of BP, 1-PP, and 6′-OH-BP are shown in Figure 2, and the pharmacokinetic parameters are presented in Table 1. The formation ratios of 6′-OH-BP from BP in the PCW and PCW-BX groups were 0.28 ± 0.09 and 0.52 ± 0.22, respectively. The formation ratios of 1-PP from BP in the PCW and PCW-BX groups were 0.17 ± 0.07 and 0.18 ± 0.04, respectively. Compared with that in the control group, the formation ratio of 6′-OH-BP from BP did not significantly change (*P* > 0.05) in the PCW group and increased by 86% (*P* > 0.05) after coadministration of BX. The formation ratio of 1-PP from BP decreased by 43% (*P* < 0.05) in the RCW group and slightly increased (*P* > 0.05) by coadministration of BX.

### 3.3. Effects of RCW and RCW-BX on Hepatic CYP3A Activity* Ex Vivo*

**Figure 3:**
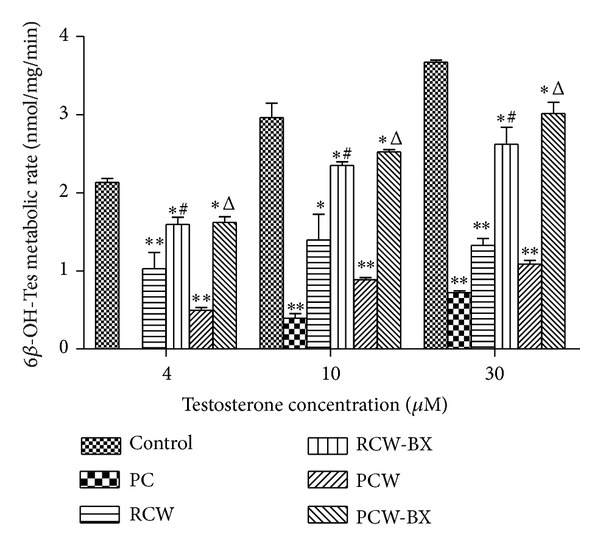
Formation rates of 6*β*-OH-Tes from Tes in rat liver microsomes (RLMs). Before the RLMs were prepared, rats were pretreated with RCW (0.6 g·kg^−1^·d^−1^, p.o.), RCW-BX (1 : 1, 0.6 g·kg^−1^·d^−1^, p.o.), PCW (0.6 g·kg^−1^·d^−1^, p.o.), PCW-BX (1 : 1, 0.6 g·kg^−1^·d^−1^, p.o.), or saline once daily for 7 d. The rats were pretreated with SKF-525A hydrochloride (0.1 g·kg^−1^, 7 d, p.o.) as the positive control (PC) for CYP3A inhibition. *P < 0.05 and **P < 0.01 compared with the control group, ^#^ *P* < 0.05 compared with the RCW group, and ^Δ^ *P* < 0.05 compared with the PCW group. Data are expressed as mean ± SD.

The hepatic CYP3A activity observed in RLMs after 7 d of pretreatment with RCW (0.6 g·kg^−1^·d^−1^, p.o.), RCW-BX (1 : 1, 0.6 g·kg^−1^·d^−1^, p.o.), or saline is shown in Figure 3. Compared with that in the control group, the formation rates of 6*β*-OH-Tes from 4, 10, and 40 *μ*mol·L^−1^ Tes in the RCW group decreased by 52% (*P* < 0.01), 53% (*P* < 0.01), and 64% (*P* < 0.01), respectively. In the RCW-BX group, the formation rates of 6*β*-OH-Tes from the same concentrations of Tes decreased by 25% (*P* < 0.05), 21% (*P* < 0.05), and 29% (*P* < 0.05), respectively. The formation rates of 6*β*-OH-Tes from 4, 10, and 40 *μ*mol·L^−1^ Tes increased by 55% (*P* < 0.05), 68% (*P* < 0.05), and 98% (*P* < 0.05), respectively, after coadministration of BX.

### 3.4. Effects of PCW and PCW-BX on Hepatic CYP3A Activity* Ex Vivo*

The hepatic CYP3A activity observed in RLMs after 7 d of pretreatment with PCW (0.6 g·kg^−1^·d^−1^, p.o.), PCW-BX (1 : 1, 0.6 g·kg^−1^·d^−1^, p.o.), or saline is shown in Figure 3. Compared with that in the control group, the formation rates of 6*β*-OH-Tes from 4, 10, and 40 *μ*mol·L^−1^ Tes in the PCW group decreased by 77% (*P* < 0.01), 70% (*P* < 0.01), and 70% (*P* < 0.01), respectively; by contrast, in the PCW-BX group, the formation rates of 6*β*-OH-Tes from the same concentrations of Tes decreased by 24% (*P* < 0.05), 15% (*P* < 0.05), and 18% (*P* < 0.05), respectively. The formation rates of 6*β*-OH-Tes from 4, 10, and 40 *μ*mol·L^−1^ Tes increased by 227% (*P* < 0.05), 185% (*P* < 0.05), and 178% (*P* < 0.05), respectively, after coadministration of BX.

### 3.5. Effects on Hepatic CYP3A Protein and mRNA Expression Levels

**Figure 4:**
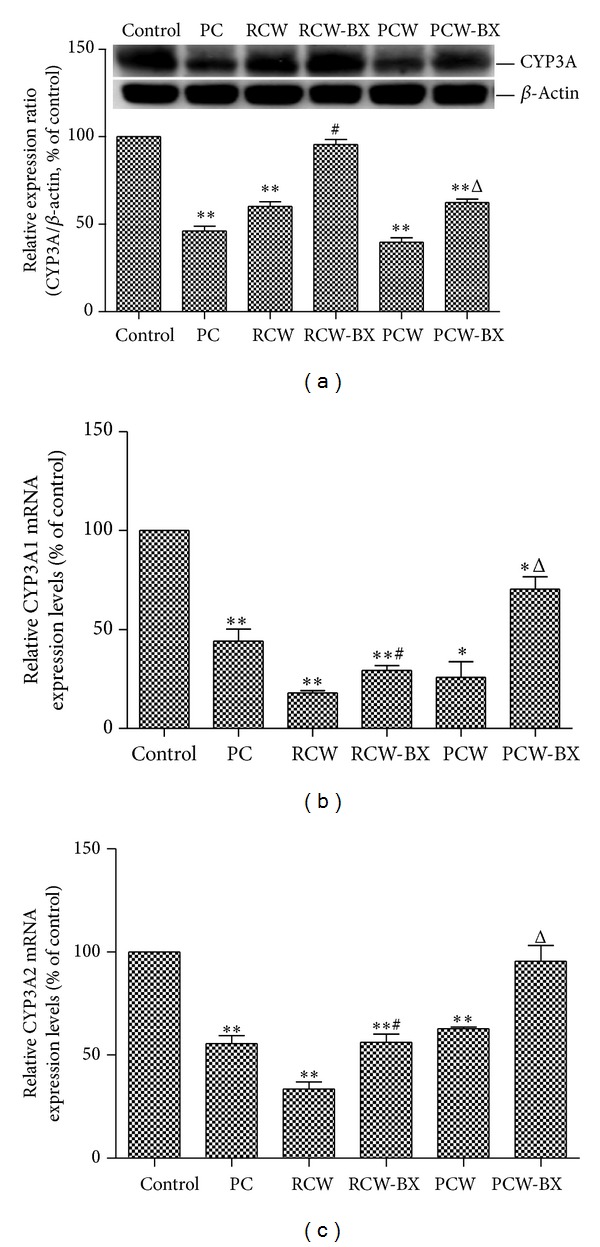
CYP3A protein (a), CYP3A1 mRNA (b), and CYP3A2 mRNA (c) expression levels in rat livers. Before the rat livers were prepared, rats were pretreated with the studied drugs or saline once daily for 7 d. The rats were pretreated with SKF-525A hydrochloride (0.1 g·kg^−1^, 7 d, p.o.) as the positive control (PC) for CYP3A inhibition. *P < 0.05 and **P < 0.01 compared with the control group, ^#^ *P* < 0.05 compared with the RCW group, and ^Δ^ *P* < 0.05  compared with the PCW group. Data are expressed as mean ± SD.

Figure 4 shows hepatic CYP3A protein (a), CYP3A1 mRNA (b), and CYP3A2 mRNA (c) expression levels in rat livers after 7 d of pretreatment with RCW (0.6 g·kg^−1^·d^−1^, p.o.), RCW-BX (1 : 1, 0.6 g·kg^−1^·d^−1^, p.o.), PCW (0.6 g·kg^−1^·d^−1^, p.o.), PCW-BX (1 : 1, 0.6 g·kg^−1^·d^−1^, p.o.), or saline. CYP3A protein expression levels were determined by measuring the ratio of CYP3A concentration to *β*-actin concentration, and CYP3A1 and CYP3A2 mRNA expression levels were determined by measuring the ratio of CYP3A1 and CYP3A2 to GAPDH, respectively. Compared with the control group, the CYP3A protein expression levels of RCW and PCW groups decreased by 40% (*P* < 0.01) and 60% (*P* < 0.01), respectively, and increased by 59% (*P* < 0.05) and 57% (*P* < 0.05) by coadministration of BX. The CYP3A1 mRNA expression levels of RCW and PCW decreased by 82% (*P* < 0.01) and 74% (*P* < 0.01), respectively, and increased by 64% (*P* < 0.05) and 172% (*P* < 0.05) after coadministration of BX. The CYP3A2 mRNA expression levels of RCW and PCW also decreased by 67% (*P* < 0.01) and 37% (*P* < 0.01), respectively, and increased by 68% (*P* < 0.05) and 52% (*P* < 0.05) after coadministration of BX.

## 4. Discussion

In the current study, we systematically investigated the influence of CW water extracts on CYP3A* in vivo* and* ex vivo*, as well as studying the changes in CYP3A activity after coadministration of BX. This investigation assesses the safety of coadministration of CW with other CYP3A-metabolizing drugs from the perspective of CYPs and probes the underlying compatibility mechanism of the two herbs.

In Chinese clinics, CW is commonly coadministered with other herbs for 7 d to treat diseases. Thus, pretreatment of rats with CW alone or coadministered with BX was conducted for 7 d in the current study. The pharmacokinetic results obtained in this study revealed that theformation ratios of 6′-OH-BP and 1-PP from BP significantly decreased in the RCW and PCW groups, respectively (Table 1, Figures 1 and 2), which demonstrates that pretreatment with RCW or PCW water extracts for 7 d can inhibit CYP3A activity* in vivo*. However, the ratios of 1-PP from BP in the RCW-treated group and those of 6′-OH-BP from BP in the PCW-treated group showed no significant variations (*P* > 0.05). We studied the influences of RCW and PCW water extracts on CYP3A activity* ex vivo* using testosterone metabolism in RLMs. The formation ratio of 6*β*-OH-Tes from Tes significantly decreased. The* ex vivo* data showed that both RCW and PCW can significantly inhibit CYP3A activity and that this inhibition is more evident* ex vivo* than* in vivo*.

Interestingly, compared with the rats orally administered RCW or PCW alone, the rats coadministered BX showed a trend of increasing metabolite formation ratios from BP, albeit insignificantly, because of considerable standard deviation (Table 1). We believe that this effect* in vivo* may be mainly attributed to the use of only five rats to investigate the pharmacokinetic behaviors of BP in each group. We further studied the effects of BX on CW-inhibited CYP3A activity via testosterone metabolism in RLMs* ex vivo*. The data displayed in Figure 3 show that the formation rates of 6*β*-OH-Tes from Tes in rats coadministered BX are statistically significantly higher than those of the rats treated with RCW or PCW alone, which supports the conclusion that inhibition of CYP3A activity caused by RCW or PCW could be reversed by BX. Moreover, RCW and PCW significantly downregulated CYP3A protein and mRNA expression levels (Figure 4), which can also be reversed by coadministration of BX. Thus, CYP3A activity decreases because of the downregulation of CYP3A protein and mRNA expression levels.

Multiherb therapy is one of the most important characteristics in Chinese clinical practice. Multiherb therapy refers to the combination of two or more herbs based on the clinical settings and properties of each herb, particularly toxic herbs. The herbs are necessarily used in combination with other prescription herbs to counteract their toxicities or side effects and simultaneously enhance therapeutic effects. CW, a representative toxic herbal medicine, has been used in China as an essential drug for 2,000 years. According to traditional Chinese medicine theory, CW is used only after processing and commonly coadministered with other herbal medicines because of its high toxicity. Historical literature and modern clinical reports indicate that BX is often coadministered with CW for treating many chronic and complicated diseases. Previous studies showed that BX can affect the content of diterpenoid alkaloids in a decoction. Processed BX can promote the transformation of diterpenoid alkaloids and decrease their content. However, given that diterpenoid alkaloids are also the pharmacological compounds in CW, content reduction cannot be used to explain the improvement in pharmacological effect. Stronger evidence must be obtained to explain the compatibility mechanism underlying the effects of CW and BX.

CYP3A plays a very important role in the metabolism of drugs. The toxicity and efficacy of drugs are closely correlated with CYP3A activity. CYP3A is the major enzyme involved in the metabolism of the active/toxic components of CW [24, 25]. In this study, we found that 7 d of CW pretreatment can significantly decrease CYP3A activity, which implies that DDIs between CW and other CYP3A-metabolizing drugs probably occur. Considerable attention must be given in clinical practice. We also examined the variations in CYP3A activity when CW was administered jointly with BX in rats and found that BX can reverse CYP3A activity inhibition, which indicates that BX can reduce the potential risk of DDIs between CW and other drugs. This finding further supports the mechanism of compatibility between CW and BX.

Besides BX, CW is frequently coadministered with other conventional herbs in clinics. Given that CW significantly inhibits CYP3A activity, the clinical use of CW-related formulae should be monitored carefully. The effects of other herbs coadministered with CW on CYP3A must be studied in future research.

## 5. Conclusions

Both RCW and PCW can significantly inhibit CYP3A activity. Such an inhibitory effect is due to the downregulation of CYP3A protein and mRNA expression levels. However, coadministration of BX can reverse CYP3A activity and expression. This activity may be one of the compatibility mechanisms between CW and BX.

## Figures and Tables

**Table 1:** Pharmacokinetic parameters of BP, 1-PP, and 6′-OH-BP in rats after intravenous injection of 0.5 mg*·*kg^−1^ BP*·*HCl. Prior to intravenous injection of BP*·*HCl, the rats were pretreated with RCW (0.6 g*·*kg^−1^ *·*d^−1^, p.o.), RCW-BX (1 : 1, 0.6 g*·*kg^−1^ *·*d^−1^, p.o.), PCW (0.6 g*·*kg^−1^ *·*d^−1^, p.o.), PCW-BX (1 : 1, 0.6 g*·*kg^−1^ *·*d^−1^, p.o.), or saline once daily for 7 d. The data presented in this figure indicate mean ± SD (*n* = 5).

Variable	Control	RCW	RCW-BX	PCW	PCW-BX
BP					
AUC_0–*t*_ (min*·*ng/mL)	14774.65 ± 2767.74	16476.25 ± 5418.23	15952.76 ± 5556.93	14550.41 ± 2813.67	13376.59 ± 1863.01
*C*_max⁡_ (ng/mL)	681.16 ± 253.80	642.29 ± 260.90	621.12 ± 215.51	607.87 ± 75.20	559.81 ± 125.62
*t*_1/2_ (min)	118.15 ± 9.83	132.44 ± 28.65	136.10 ± 16.95	142.66 ± 7.40	143.06 ± 20.71
CL (mL/min/kg)	34.37 ± 6.05	33.89 ± 15.59	33.97 ± 11.66	34.53 ± 7.23	37.28 ± 4.77
*V*_*d*_ (L/kg)	5806.61 ± 752.26	6529.71 ± 3383.06	6587.34 ± 2071.05	7160.69 ± 1862.87	7716.18 ± 1609.23
6′-OH-BP					
AUC_0–*t*_ (min*·*ng/mL)	3515.02 ± 1032.97	1569.88 ± 873.69	3006.41 ± 1559.71	4078.18 ± 1400.67	7175.93 ± 3818.45
*C*_max⁡_ (ng/mL)	38.96 ± 17.86	14.91 ± 6.62	30.03 ± 14.00	37.93 ± 7.15	57.20 ± 24.21
*t*_max⁡_ (min)	43.62 ± 11.61	82.16 ± 31.02	58.45 ± 14.63	53.95 ± 24.71	68.24 ± 15.09
*t*_1/2_ (min)	42.00 ± 26.83	36.00 ± 13.42	48.00 ± 16.43	42.00 ± 16.43	42.00 ± 16.43
CL (mL/min/kg)	151.43 ± 39.84	468.80 ± 386.76	203.51 ± 90.24	138.68 ± 59.68	83.87 ± 34.23
*V*_*d*_ (L/kg)	9667.04 ± 4169.74	64877.77 ± 79892.21	17018.80 ± 9489.70	10046.30 ± 3828.53	7761.37 ± 2356.87
6′-OH-BP/BP	0.24 ± 0.06	0.10 ± 0.05*	0.18 ± 0.04	0.28 ± 0.09	0.52 ± 0.22
1-PP					
AUC_0–*t*_ (min*·*ng/mL)	4249.31 ± 1290.50	3991.68 ± 1627.14	2272.67 ± 857.32	2459.69 ± 823.79	2356.85 ± 456.45
*C*_max⁡_ (ng/mL)	27.35 ± 12.57	17.05 ± 6.81	11.60 ± 3.09	11.11 ± 2.23	10.66 ± 0.27
*t*_max⁡_ (min)	160.16 ± 52.85	200.74 ± 72.28	135.10 ± 23.37	186.09 ± 104.57	176.38 ± 33.75
*t*_1/2_ (min)	48.00 ± 26.83	60.00 ± 30.00	54.00 ± 13.42	60.00 ± 21.21	84.00 ± 13.42
CL (mL/min/kg)	126.20 ± 36.57	136.69 ± 71.03	248.13 ± 96.14	209.21 ± 74.58	218.34 ± 40.08
*V*_*d*_ (L/kg)	30074.20 ± 16188.54	34986.74 ± 11950.65	46189.83 ± 28505.89	49035.59 ± 22809.86	55990.95 ± 16636.48
1-PP/BP	0.30 ± 0.11	0.25 ± 0.08	0.15 ± 0.05^∗#^	0.17 ± 0.07*	0.18 ± 0.04*

6′-OH-BP/BP means AUC_0–*t*_ of 6′-OH-BP/AUC_0–*t*_ of BP; 1-PP/BP means AUC_0–*t*_ of 1-PP/AUC_0–*t*_ of BP. *P < 0.05 compared with control group. ^#^ *P* < 0.05 compared with RCW group.
